# Improvement of Fermi-Level Pinning and Contact Resistivity in Ti/Ge Contact Using Carbon Implantation

**DOI:** 10.3390/mi13010108

**Published:** 2022-01-10

**Authors:** Iksoo Park, Donghun Lee, Bo Jin, Jungsik Kim, Jeong-Soo Lee

**Affiliations:** 1 Department of Electrical Engineering, Pohang University of Science and Technology (POSTECH), Pohang 37673, Korea; isparkk@postech.ac.kr (I.P.); leedh009@postech.ac.kr (D.L.); shengzhi86@postech.ac.kr (B.J.); 2 Research and Development Department, Innovative General Electronic Sensor Technology Co., Ltd. (IGEST), Pohang 37673, Korea; 3 Department of Electrical Engineering, Gyeongsang National University, Jinju 52828, Korea; jungsik@gnu.ac.kr

**Keywords:** MS contact, fermi-level pinning, titanium, germanide, carbon, implantation

## Abstract

Effects of carbon implantation (C-imp) on the contact characteristics of Ti/Ge contact were investigated. The C-imp into Ti/Ge system was developed to reduce severe Fermi-level pinning (FLP) and to improve the thermal stability of Ti/Ge contact. The current density (*J*)-voltage (*V*) characteristics showed that the rectifying behavior of Ti/Ge contact into an Ohmic-like behavior with C-imp. The lowering of Schottky barrier height (SBH) indicated that the C-imp could mitigate FLP. In addition, it allows a lower specific contact resistivity (*ρ_c_*) at the rapid thermal annealing (RTA) temperatures in a range of 450–600 °C. A secondary ion mass spectrometry (SIMS) showed that C-imp facilitates the dopant segregation at the interface. In addition, transmission electron microscopy (TEM) and electron energy loss spectroscopy (EELS) mapping showed that after RTA at 600 °C, C-imp enhances the diffusion of Ge atoms into Ti layer at the interface of Ti/Ge. Thus, carbon implantation into Ge substrate can effectively reduce FLP and improve contact characteristics.

## 1. Introduction

As a channel material for the next-generation field-effect transistors (FETs), Germanium (Ge) is considered a promising alternative to silicon (Si) owing to its higher carrier mobility and the process compatibility with the advanced Si microfabrication. However, the low-solid solubility and the high-diffusion coefficient of n-type dopants in Ge hinder the realization of low specific contact resistivity (*ρ_c_*) [1]. Moreover, Fermi-level pinning (FLP) caused by the metal-induced gap states (MIGS) at the metal/Ge interface is another problem to be solved [2,3,4,5]. FLP strongly occurs near the Ge valence band (*E_v_*) and forces the electron Schottky barrier height (e-SBH) above 0.5 eV irrespective of the metal workfunction [6]. Several approaches, including dopant segregation [7], dipole formation [8], and surface treatment [9] were proposed to mitigate FLP phenomena. Recently, the use of an ultra-thin insulator between the metal and Ge showed an effective reduction of FLP but the degradation of *ρ_c_* due to a high tunneling resistance [10,11,12,13]. The formation of metal germanide can be another approach because the MIGS from metal dangling bond states in germanide can lead to an FLP reduction [14,15].

Ion implantation is another approach to achieving low *ρ_c_* and suppressing dopant-diffusion behaviors. For example, Germanium implantation before silicidation induces surface amorphization to aid an epitaxial regrowth on the semiconductor surface [16]. Carbon implantation (C-imp) has been introduced in Ni-silicide and Ni-germinide contacts to reduce contact resistivity [17,18]. However, Ti/Ge contact with carbon implantation has been rarely reported.

Here, we investigated the effects of C-imp on the FLP reduction of a Ti/Ge contact and the related contact characteristics. Electrical characteristics were measured using the multiring-circular transmission line model (MR-CTLM) structure and Schottky barrier diode (SBD). Physical and structural properties of Ti/Ge contact with C-imp were analyzed using scanning electron microscopy (SEM), transmission electron microscopy (TEM), electron energy loss spectroscopy (EELS), and secondary ion mass spectrometry (SIMS).

## 2. Materials and Methods

N-type Ge wafers moderately doped with phosphorus (~10^18^ cm^−3^) were cleaned in a 1:100 diluted HF (dHF) solution and deionized (DI) water to remove native oxide. Subsequently, C^+^ ions were implanted into the Ge substrate at a dose of 1 × 10^15^ cm^−2^ and an implantation energy of 10 keV. A reference sample without C-imp was also prepared. A SBD of Ti/Ge structure and a MR-CTLM structure were fabricated on the Ge substrate to characterize electrical properties. First, a 100 nm thick SiO_2_ was deposited to isolate the contact holes using a plasma-enhanced chemical vapor deposition (PECVD). Then, the metal contact was formed using the conventional photolithography process. Sequentially, the oxide was etched using a dry etcher, and a Ti (5 nm)/TiN (5 nm) was deposited using a DC sputtering system. After a metal lift-off process, rapid thermal annealing (RTA) was performed in N_2_ ambient for 60 s at 450–600 °C. Finally, a 100 nm thick Al was deposited as contact pad metal. The electrical measurements of current (*I*)–bias voltage (*V*) were performed using Keithley 4200-SCS. TEM images of the Ti/Ge structure without and with C-imp were obtained using a *JEOL JEM 2200FS* with an image Cs-corrector.

## 3. Results

**Figure 1:**
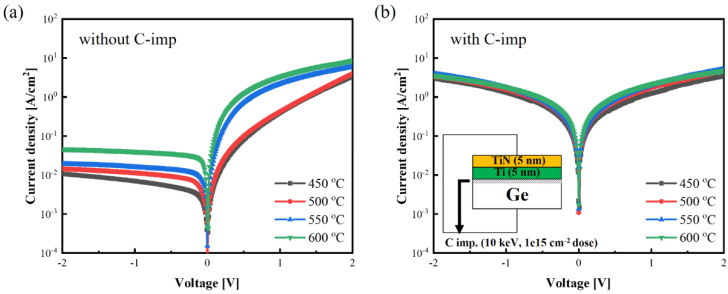
*J*-*V* characteristics of the Ti/Ge contact (**a**) without and (**b**) with C-imp at RTA temperatures in a range of 450–600 °C for 60 s in N_2_ ambient.

Figure 1 shows the *J*-*V* characteristics of the Ti/Ge contacts with and without C-imp at RTA temperatures in a range of 450–600 °C for 60 s in N_2_ ambient. The Ti/Ge contact without C-imp shows a typical rectifying behavior attributed to a strong FLP near the *E_v_*, which leads to a significantly high e-SBH and reduces the reverse current density. On the other hand, the Ti/Ge contact with C-imp shows an Ohmic-like behavior with relatively high current density under the reverse regime, indicating the alleviation of FLP. 

**Figure 2:**
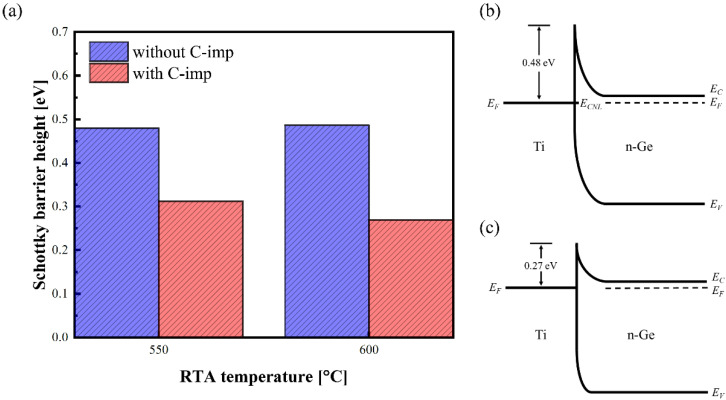
(**a**) e-SBHs of the Ti/Ge contacts without (blue box) and with (red box) C-imp after RTA at 550 °C and 600 °C for 60 s in N_2_ ambient, respectively. Schematics of energy band diagrams for Ti/Ge contact (**b**) without and (**c**) with C-imp after RTA at 600 °C.

Figure 2a shows the extracted e-SBHs of the Ti/Ge contacts without (blue box) and with (red box) C-imp after RTA at 550 °C and 600 °C, respectively, for 60 s in N_2_ ambient. The e-SBHs were extracted from the current-temperature (*I*-*T*) curves in a range of 300–378 K. The *I*-*V* relationship of a Schottky barrier diode is represented by [19]

(1) $$I=AA^{*}T^{2}e^{-q\emptyset_{B}/kT}\left(e^{qV/nkT}-1\right)=I_{S1}e^{-q\emptyset_{B}/kT}\left(e^{qV/nkT}-1\right)=I_{S}\left(e^{qV/nkT}-1\right)$$

where *I_s_* is the saturation current, *A* is the diode area, A* = 4*πqk*^2^m*/*h*^3^ = 120 (m*/*m*) A/cm^2^∙K^2^ Richardson’s constant, *Φ_B_* is the barrier height, and *n* is the ideality factor. For V≫kT/q Equation (1) can be written as follows:

(2) $$\ln\left(I/T^{2}\right)=\ln\left({\mathrm{AA}}^{*}\right)-q\left(\emptyset_{B}-V/n\right)/kT$$

(3) $$\emptyset_{B}=\frac{V}{n}-\frac{k}{q}\frac{d\left[\ln\left(I/T^{2}\right)\right]}{d\left(1/T\right)}=\frac{V}{n}-\frac{2.3k}{q}\frac{d\left[\log\left(I/T^{2}\right)\right]}{d\left(1/T\right)}$$

Therefore, the barrier height is calculated from the slope (=*d*[ln(I/*T*^2^)]/*d*(1/*T*)). The bandgap and electron affinity in eV of Ge at 300 K are 0.66 and 4.0 eV, respectively. The workfunction of Ti metals is about 4.3 eV. When Fermi level is pinned near *E_v_* of Ge, *Φ_B_* of ~0.6 eV is calculated. If there is negligible FLP, *Φ_B_* of ~0.3 eV is obtained.

Without C-imp, the SBH of ~0.48 eV was obtained for both 550 °C and 600 °C RTA, indicating the occurrence of FLP. In contrast, the SBH with C-imp was significantly reduced from 0.31 eV at 550 °C to 0.27 eV at 600 °C.

Figure 2b,c show schematics of the energy band diagrams for Ti/Ge contacts. Without C-imp, Fermi-level on the Ti side is pinned with the charge neutrality level (*E_CNL_*) due to FLP [6]. 

**Figure 3:**
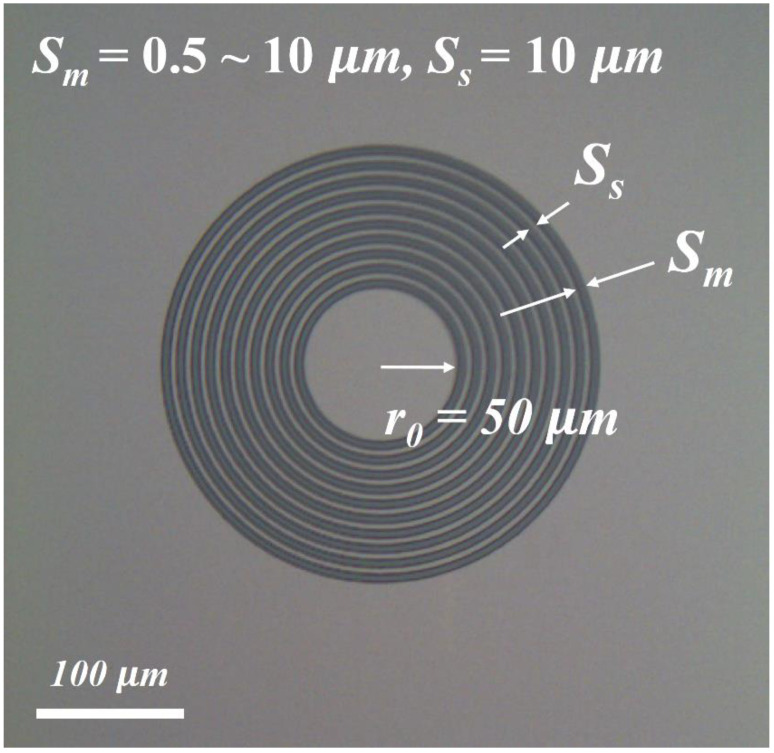
Top-view SEM image of the fabricated MR-CTLM structure.

Figure 3 shows a top-view SEM image of the fabricated MR-CTLM structure to extract *ρ_c_* and the sheet resistance beneath the metal (*R_S_*). The current flows through multiple metal-semiconductor structures from the center region to the outer-circle region. From the *I*-*V* curve of MR-CTLM, the total resistance (*R_tot_*) is expressed as the sum of the effective resistance (*R_eff_*) and the parasitic resistance (*R_pr_*) as follows [20]:

(4) $$R_{tot}=R_{eff}+R_{pr}$$

(5) $$R_{eff}=\frac{R_{s}}{2\pi }\sum_{i=0}^{9}\left[\ln\left(\frac{r_{i}+S_{m}}{r_{i}}\right)+L_{t}\left(\frac{1}{r_{i}}+\frac{1}{r_{i}+S_{m}}\right)\right]$$

(6) $$R_{pr}=\frac{R_{m}}{2\pi }\left[\sum_{i=1}^{9}\ln\left(\frac{r_{i}-L_{t}}{r_{i}-S_{s}+L_{t}}\right)\right]$$

where *r*_0_*~r*_9_ are the inner radius of the serial CTLM. *S_m_* and *S_s_* are the spacing among metal rings and dielectric rings, respectively. *L_t_* is the transfer length. *S_s_* = 10 μm, *r*_0_ = 50 μm, and *S_m_*, from 0.5 to 10 μm were defined using an i-line stepper. *ρ_c_* was calculated from the *L_t_* ($=\sqrt{\rho_{c}/R_{s}}$) which was extracted by fitting a set of *R_t_*-*S_m_* data using Equations (4)–(6).

**Figure 4:**
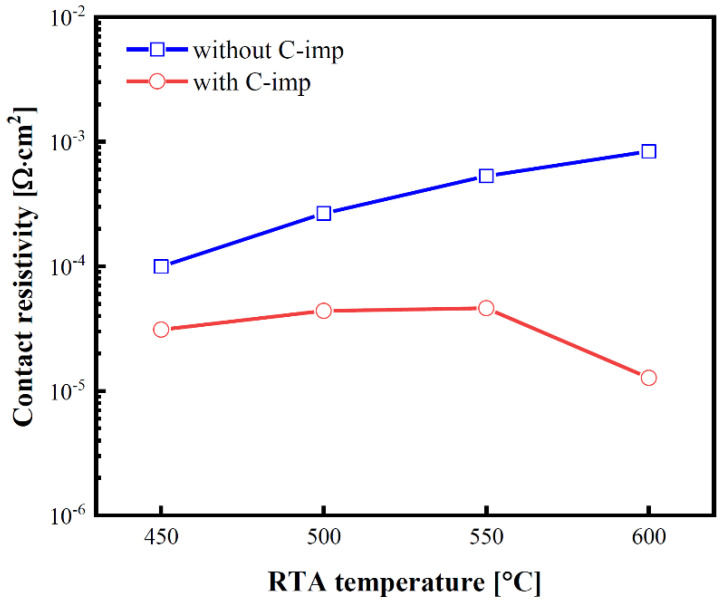
*ρ_c_* of the Ti/Ge contacts without (blue curve) and with (red curve) C-imp as a function of RTA temperatures ranging from 450 to 600 °C.

Figure 4 shows the extracted *ρ_c_* values versus RTA temperature. *ρ_c_* was obtained using a MR-CTLM test structure [20]. A relatively high *ρ_c_* value seems mainly because of the low activation of a substrate doping concentration of ~1 × 10^18^ cm^−3^ [21,22]. After RTA annealing at 600 °C, the *ρ_c_* values of the Ti/Ge with and without C imp were 1.3 × 10^−5^ and 8.4 × 10^−4^ Ω∙cm^2^, respectively. Owing to the FLP effect, the Ti/Ge contact without C-imp shows *ρ_c_* values higher than 1.0 × 10^−4^ Ω∙cm^2^.

To further analyze the effect of C-imp on the Ti/Ge composition, TEM and SIMS were conducted. The decrease of *ρ_c_* is mainly attributed to the dopant segregation in the Ti/Ge interface [23]. In particular, for the Ti/Ge contact with C-imp after RTA at 600 °C, a further reduction of *ρ_c_* is observed. These results can be expected by TiGe_x_ formation. The low resistive C54-TiGe_x_ is formed at a temperature above 600 °C [24], which mitigates FLP and improves the contact resistivity [14,15].

**Figure 5:**
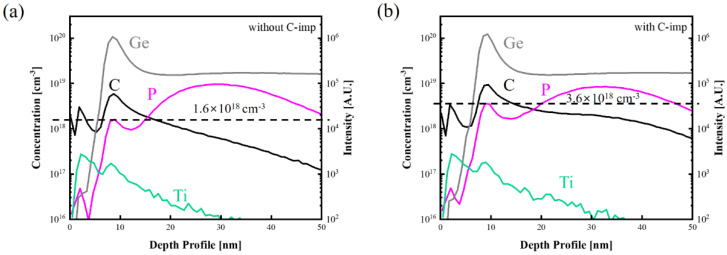
SIMS profiles for Ti/Ge contacts (**a**) without and (**b**) with C-imp after RTA at 600 °C. With C-imp, a dopant (phosphorous) segregation at the Ti/Ge interface is clearly observed.

Figure 5a,b show SIMS profiles for Ti/Ge contacts without and with C-imp, respectively. At the Ti/Ge interface with C-imp, the peak P concentration increases from 1.6 × 10^18^ cm^−3^ to 3.6 × 10^18^ cm^−3^, attributed to the dopant segregation facilitated by carbon [18]. This dopant segregation can increase the tunneling current by reducing the depletion thickness at the interface and lowering the contact resistivity.

**Figure 6:**
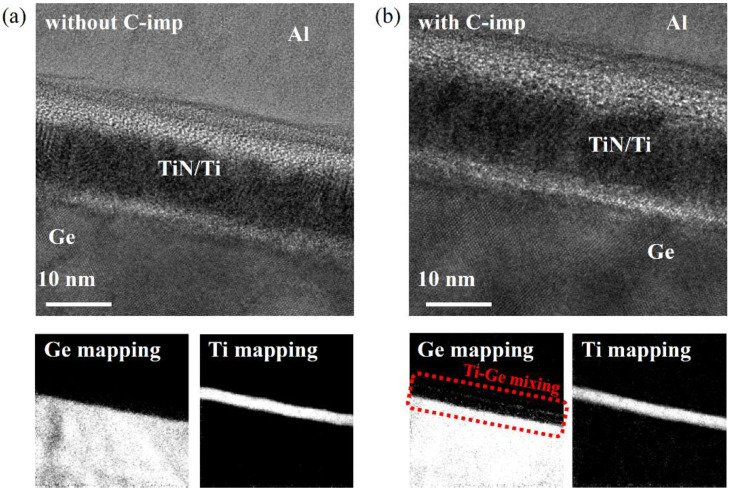
Cross-sectional TEM images and corresponding electron energy loss spectroscopy (EELS) mapping images for Ge and Ti in the Ti/Ge contacts (**a**) without and (**b**) with C-imp after RTA at 600 °C.

To directly observe the microstructure of Ti/Ge contact, the cross-sectional TEM images and the corresponding EELS were analyzed. The samples were prepared after RTA at 600 °C for 60 s in N_2_ ambient, as shown in Figure 6. In EELS maps, a bright region represents the area that the element of interest is abundant. With C-imp, Ge element is considerably observed in the Ti layer (red box in Figure 6b). The diffused Ge reacts with Ti and forms the Ti-germanide during the RTA process, which is beneficial to reduce the contact resistivity [14,15]. These results show that the C-imp is a promising approach to lower the contact resistivity in Ti/Ge contact by inducing the dopant segregation and Ge diffusion into the Ti layer.

## 4. Conclusions

We investigated the electrical and material characteristics of a Ti/Ge contact with C-imp. The current-voltage behavior shows that the carbon implantation changes the Ti/Ge rectifying behavior into an Ohmic-like behavior above RTA at 450 °C. The extracted Schottky barrier height was also decreased due to the mitigation of Fermi-level pinning. The specific contact resistivity of the Ti/Ge contact with C-imp was significantly reduced by approximately two orders of magnitude. Transmission electron microscopy and secondary ion mass spectrometry showed that carbon element at the Ti/Ge interface facilitates the dopant segregation and induces the diffusion of Ge into Ti layer. Therefore, the carbon implantation is promising to improve the Ti/Ge contact properties for high-performance Ge-FET applications.
